# Applied Open Science for Secondary Data Analysis and Meta-Analysis

**DOI:** 10.1093/geroni/igaa057.1880

**Published:** 2020-12-16

**Authors:** Jennifer Lodi-Smith

**Affiliations:** Canisius College, Buffalo, New York, United States

## Abstract

This talk will provide guidance on the practicalities of open science for secondary data analysis and meta-analyses. Example studies will provide practical considerations for preregistering complex projects, insights into strategies for transparently reporting deviations from preregistrations, advice on deciding when and how to share sensitive data, and tips on transparent documentation of analysis code. Examples will be drawn from an ongoing meta-analysis of the relationship between self-concept clarity and self-esteem (https://osf.io/sa2bx/), the Rochester Adult Longitudinal Study (https://osf.io/ya4ph/), and the Aging and Autism Study (https://osf.io/g9c3e/). The pedagogical value of preregistration will be emphasized throughout the talk.

